# A Swiss flag through the chest: a case report of a rare transmediastinal impalement

**DOI:** 10.1097/RC9.0000000000000873

**Published:** 2026-08-21

**Authors:** Jonathan Schumacher, Gyoergy Lang, Isabelle Opitz, Didier Schneiter

**Affiliations:** a Department of Thoracic Surgery, University Hospital Zurich, Zurich, Switzerland; b Department of Cardiothoracic Surgery, Oslo University Hospital, Oslo, Norway

**Keywords:** case report, clamshell thoracotomy, penetrating chest injury, transmediastinal injury, trauma management

## Abstract

**Introduction:**

Management of transmediastinal penetrating injuries (TMI) is challenging. We report an educational flagpole-related case.

**Case presentation:**

A 61-year-old woman arrived at our trauma center after a boating accident resulting in transthoracic impalement from a fall onto a flagpole. She was hemodynamically stable, fully conscious, and without additional injuries. Chest X-ray showed an approximately 30-cm foreign body penetrating the thorax from the left and ending paramediastinally on the right, suggesting mediastinal and pulmonary involvement. CT confirmed that the flagpole entered through the left third intercostal space (ICS), traversed the left upper lobe and mediastinum near the aortic arch and between the trachea and esophagus, and ended in the right lung. Bronchoscopy and esophagoscopy excluded endoluminal injury. A clamshell thoracotomy was performed via the fourth ICS and was extended to the flagpole entry site. The flagpole penetrated both upper lobes and the mediastinum near the aortic arch. It was carefully withdrawn, and a minor adventitial injury to the aortic arch was identified. Pulmonary lacerations and a left upper lobe segmental artery were sutured. Tracheal exploration revealed no injury. She was extubated 7 hours postoperatively, remained in the ICU for three days, and was discharged after an uneventful 18-day stay. Follow-up showed no complications.

**Clinical discussion:**

Unlike common thoracic injuries, TMIs often require surgery due to the frequent involvement of critical organs. This case illustrates that stable patients benefit from detailed CT and endoscopic assessment to guide operative strategy. Controlled flagpole shortening enabled CT imaging. Clamshell thoracotomy provided optimal exposure of all involved structures.

**Conclusion:**

This TMI case highlights the importance of cautious shortening of impaling objects that exceed CT gantry limits and the critical role of multidisciplinary trauma management.

**Graphical abstract available at:**

https://links.lww.com/IJSCR/A126

## Introduction

The management of transmediastinal penetrating injuries (TMI) is challenging, with reported mortality rates of up to 60%^[^1^]^. Although relatively rare, TMIs are most frequently caused by gunshots; however, other projectiles or even impalement can also result in these life-threatening injuries^[^2,3^]^. Unlike more common thoracic injuries, TMIs carry a higher rate of operative intervention^[^1^]^. High mortality is largely due to hemodynamic instability from thoracic vascular injury, occurring in about 50% of patients with mediastinal gunshot wounds^[^4^]^. Given the mediastinum’s dense concentration of major vessels and vital organs, penetrating injuries rarely spare critical structures, explaining the lethal nature of TMIs^[^1^]^. Improvements in trauma systems and faster transport have increased survival to hospital arrival for patients with potentially lethal injuries; nonetheless, pre-hospital mortality remains high^[^5^]^.

HIGHLIGHTS

- The management of transmediastinal penetrating injuries (TMI) is challenging and associated with high mortality. This case demonstrates the successful treatment of a complex flagpole-related TMI.
- The patient’s hemodynamic stability enabled comprehensive multidisciplinary trauma management, including bronchoscopy, esophagoscopy, and a clamshell thoracotomy for the controlled removal of the foreign body.
- Cautious shortening and continuous stabilization of a long impaling object facilitated safe CT imaging and thorough preoperative assessment.
- The clamshell thoracotomy provided excellent exposure of all potentially injured structures.

A small subset of patients with stable vital signs requires only observation. The key challenge is efficiently triaging unstable patients for emergency surgery and operating on stable patients only if the injuries are potentially fatal. Initial assessment must focus on determining the trajectory of the penetrating object, as this directly reflects the extent of underlying injury^[^6^]^. Current management relies heavily on advanced CT imaging, which precisely defines the injury path and evaluates mediastinal structures, enabling a targeted treatment approach^[^3^]^.

Herein, we report a case of a complex TMI caused by a long flagpole, focusing on trauma room (TR) management, preoperative investigations, and the surgical procedure.

This work has been reported in line with the SCARE checklist^[^7^]^.

## Case presentation

A 61-year-old woman was airlifted to our major trauma center following a boating accident resulting in transthoracic impalement by a flagpole (air distance, 43 miles). She had a history of right-sided C7/T1 foraminotomy for foraminal stenosis with C8 radiculopathy and multilevel decompression and instrumented fusion (L1-S1) for degenerative lumbar spine disease. She had no regular medications, no known allergies, and a non-contributory social and family history.

The patient slipped while transferring from one boat to another and was impaled by the flagpole. Initially, the local fire brigade cut the pole with a cold saw to free the patient for transfer. The rescue team arrived at our TR 120 minutes after the incident, bringing a stable and fully oriented patient (GCS 15, normotensive, with a normal heart rate, SpO_2_ > 92% on 4 L/min of oxygen via a face mask; 22:00). During the prehospital rescue, the patient received 1 g of intravenous tranexamic acid and continuous oxygen therapy.

**Figure 1.**
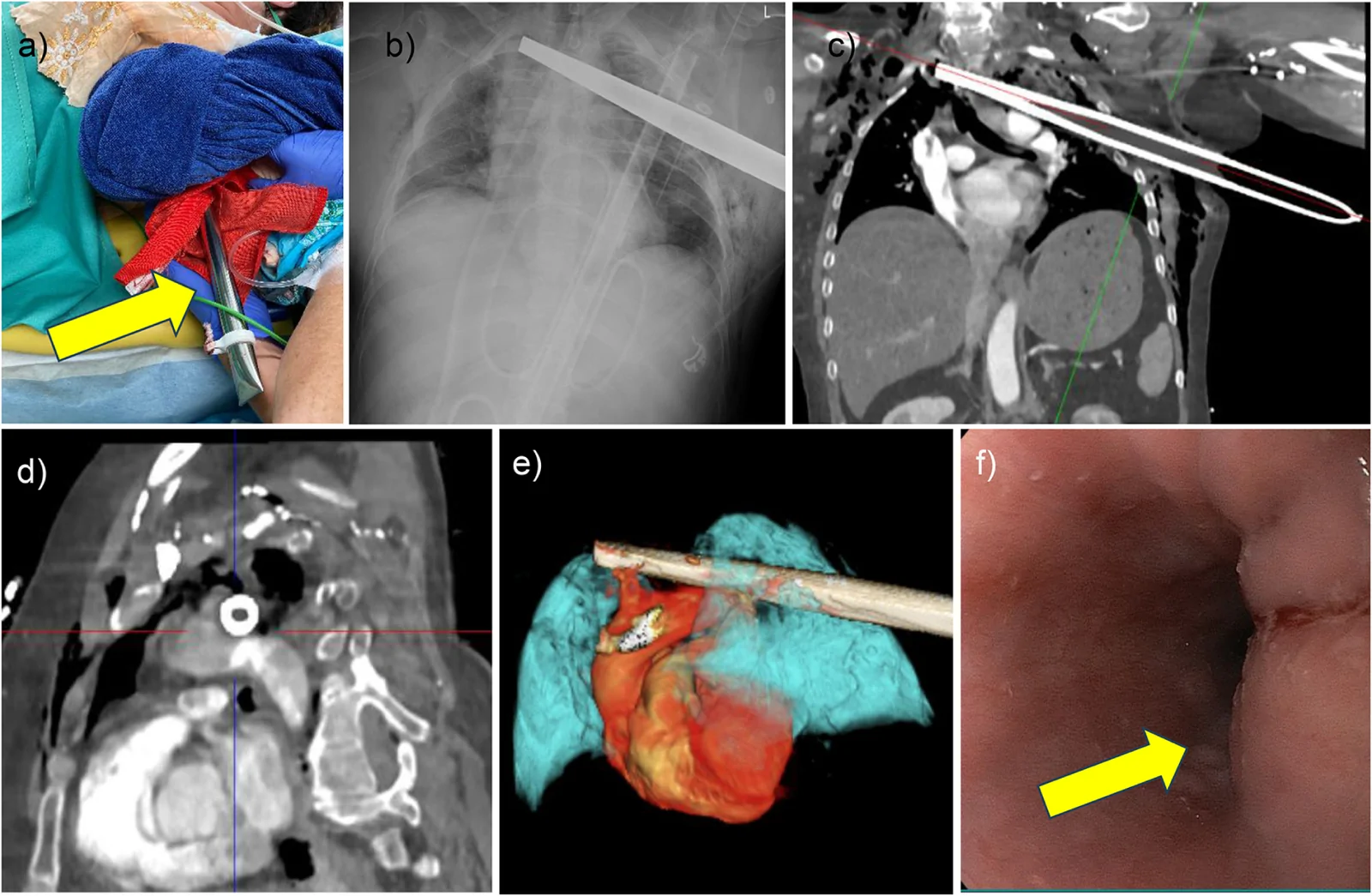
(A) Penetrating flagpole (yellow arrow) manually stabilized throughout trauma management; (B) Initial chest x-ray; (C) Coronal CT scan; (d) Sagittal CT scan; (e) Three-dimensional CT reconstruction demonstrating the position of the flagpole in relation to the lungs and aortic arch; (f) Preoperative esophagoscopy showing extrinsic compression of the ventral esophageal wall (yellow arrow) without evidence of an endoluminal lesion.

The primary survey revealed no additional injuries (Fig. 1a). On auscultation, a wheeze was noted over the left lung apex, with otherwise normal heart sounds and equal breath sounds bilaterally. Chest X-ray showed an approximately 30-cm radiopaque foreign body penetrating the left hemithorax and extending to the right side (Fig. 1b). Based on the object’s trajectory – entering very posteriorly on the left hemithorax and directed cranially toward the cervical spine – we suspected mediastinal and pulmonary injuries. Routine laboratory investigations and viscoelastic hemostatic assay (ROTEM) on arrival were unremarkable; hemoglobin, at 116 g/L, was mildly below the reference range but did not prompt transfusion.

Given the patient’s stable condition, a computed tomography (CT) scan, including a CT angiogram (CTA), was performed. For the CT scan, the remaining portion of the flagpole was shortened by the fire brigade using a cold saw. During this procedure, extreme caution was taken to stabilize the penetrating flagpole, avoiding secondary injury. CT imaging confirmed that the flagpole entered the left chest through the third ICS (Fig. 1c–e), pierced the left upper lobe, and traversed the mediastinum, narrowly missing the aortic arch inferiorly and lying close to the left subclavian artery. It then passed between the trachea and esophagus before ending in the apex of the right lung, without apparent contralateral thoracic wall injury. The CT scan also showed small bilateral pneumothoraces, consistent with the soft tissue emphysema seen on the initial chest X-ray.

**Figure 2.**
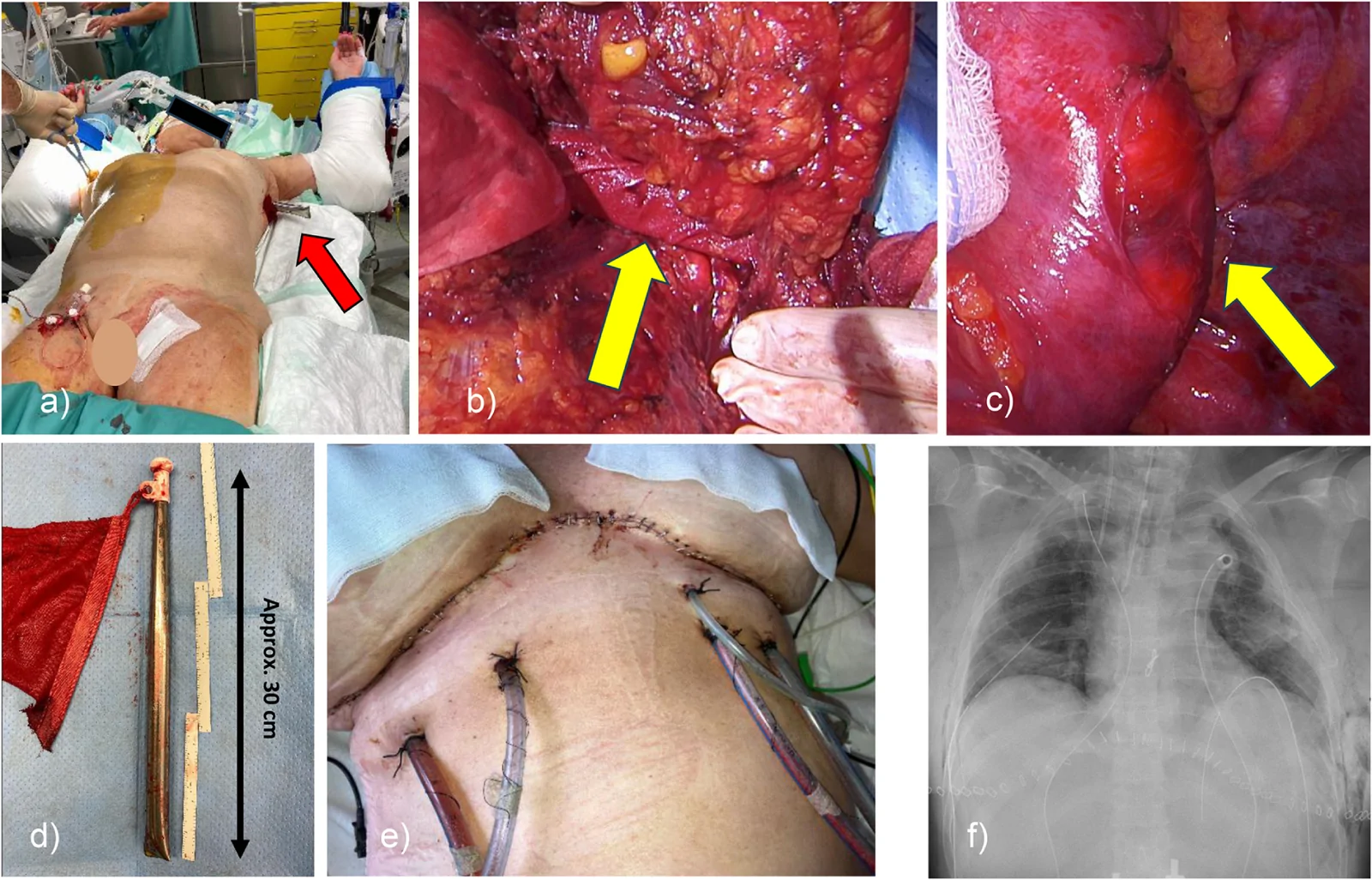
(a) The patient was positioned supine with both arms abducted; The red arrow indicates the shortened flagpole; Double-lumen endotracheal intubation was performed; right-sided femoral arterial and venous access was established for rapid cardiopulmonary bypass if required; and skin preparation was performed using a povidone-iodine solution. (b) Intraoperative image showing the flagpole and surrounding flag tissue (yellow arrow) during chest wall penetration; the left lung is on the left side of the screen. (c) Aortic wall after removal of the flagpole, showing a small adventitial laceration (yellow arrow). (d) Flagpole and flag tissue after removal. (e) Postoperative view of the surgical access, chest tubes, and vacuum drain. (f) Postoperative chest x-ray.

Since the patient remained stable and the CT scan could not definitively exclude tracheal or esophageal injury, bronchoscopy was performed via a laryngeal mask prior to tracheal intubation to permit unobstructed visualization of the tracheobronchial tree, and esophagoscopy was performed before surgery. Despite extrinsic compression on the ventral wall of the esophagus approximately 2 cm cranial to the aortic arch, and minor tracheal deviation, no endoluminal lesion was identified in either structure (Fig. 1f). To prepare for potential major hemorrhage during surgery, femoral venous and arterial catheters were inserted to enable rapid cardiopulmonary bypass if required (Fig. 2a). A double-lumen endotracheal tube was placed to facilitate operative exposure and allow single-lung ventilation if required. The flagpole was stabilized throughout pre-hospital care until the start of surgery.

Surgery was performed by two senior consultant thoracic surgeons, assisted by a thoracic surgery resident. A clamshell incision was made in the fourth ICS and extended to the flagpole entry point. As suspected from CT imaging, the flagpole had penetrated both upper lobes and traversed the mediastinum just above the aortic arch (Fig. 2b). The flagpole was carefully removed under direct vision. A minor adventitial injury to the aortic arch was identified (Fig. 2c and d; Supplemental Digital Content available at: https://links.lww.com/IJSCR/A125). Given its superficial nature and absence of active bleeding, conservative management was elected. Pulmonary lacerations were repaired with 4-0 PDS continuous double-layer running sutures, and a segmental artery in the left upper lobe was oversewn with 4-0 Prolene. Bilateral tracheal exploration revealed no laceration. The right thoracic wall showed an apicoposterior contusion with a parietal pleural lesion. Four chest tubes were placed bilaterally (one basal and one apicoposterior on each side), and a subcutaneous vacuum drain was inserted (Fig. 2e and f). Operative duration was 2.5 hours. Intraoperative blood loss was 130 mL, collected via Cell Saver**®**.

Prophylactic broad-spectrum antibiotics (amoxicillin/clavulanic acid) were administered perioperatively. The patient was extubated after 7 hours and remained in the ICU for three days. Postoperatively, the patient developed tingling, neuropathic pain, and significant motor deficits affecting right-hand flexion and finger movement. These symptoms were thought to result from irritation of the inferior brachial plexus (C8–T1), likely due to the contralateral trajectory of the flagpole (Clavien–Dindo classification grade II due to pharmacological treatment)^[^8^]^. MRI demonstrated perineural edema/haematoma surrounding the right lateral and medial fascicles without evidence of nerve discontinuity. By discharge, the neurological deficits had improved substantially.

Follow-up gastroscopy on POD 1 and bronchoscopy on POD 2 confirmed no mucosal laceration. The final drain was removed on POD 8 following a persistent air leak, expected after extensive lung injury. Pharmacological thromboprophylaxis consisted of unfractionated intravenous heparin (10 000 IU/24 h) until POD 8, followed by dalteparin 5000 IU once daily, in combination with compression stockings.

**Figure 3.**
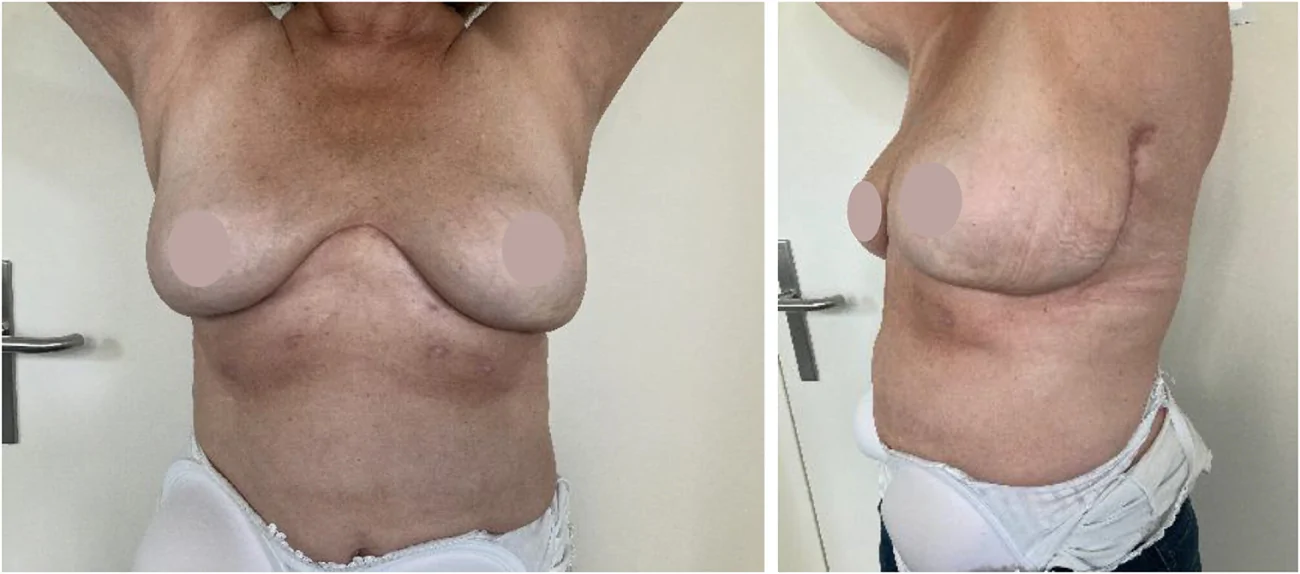
Three-month follow-up demonstrating a satisfactory clinical course.

She was discharged 18 days postoperatively to a specialized neurological rehabilitation facility (earlier transfer was limited by capacity). Follow-up at 3 months showed a satisfactory clinical and radiological course (Fig. 3). She could lift her right arm and rotate her hand, with largely normal tactile sensation. At nearly 3 years of follow-up, electrophysiological examination demonstrated progressive reinnervation of median- and radial-innervated hand and arm muscles, with persistent plegia of ulnar-innervated hand muscles. Clinically, fist formation had recovered, and neuropathic pain was controlled with pregabalin. Physiotherapy and occupational therapy are ongoing.

Table 1 provides a timeline of key events from injury to long-term follow-up.

**Table 1:** Timeline of clinical key events.

Time	Event
Prehospital	
ca. 20:00	Boating accident; flagpole impalement
20:10-21:10	On-scene rescue by the local fire brigade, water police, and emergency physician; flagpole shortened
ca. 21:10-21:24	Transfer to helicopter at local hospital’s helipad by ambulance
**Trauma Room**	
22:07	Arrival at the trauma center by helicopter; patient hemodynamically stable, GCS 15, SpO_2_ > 92% on 4 L/min O_2_
22:08	Primary survey; no additional injuries identified; chest X-ray: 30 cm radiopaque foreign body traversing left to right thorax
22:15–23:00	Flagpole shortened by fire brigade using cold saw to permit CT imaging; transfer to CT under continuous manual stabilization
23:15–00:00	CT scan: flagpole trajectory through left third ICS, left upper lobe, mediastinum near aortic arch, between trachea and esophagus, ending in right lung apex; bilateral small pneumothoraces
**Operating Room**	
00:43	Arrival at operating room and preparations for surgery
00:45	Femoral arterial and venous access for potential cardiopulmonary bypass established
02:00	Bronchoscopy via laryngeal mask: no endoluminal tracheobronchial injury
02:24	Esophagoscopy: extrinsic anterior compression without endoluminal lesion
03:18–05:51	Surgery (Clamshell thoracotomy, flagpole removed under direct vision, adventitial aortic arch laceration managed conservatively; pulmonary lacerations and left upper lobe segmental artery repaired)
**Intensive Care Unit**	
06:20	Transfer to ICU
ca. 10:00	Extubation and high-flow oxygen therapy
POD 1	Follow-up gastroscopy: no laceration; suspected irritation of the right inferior brachial plexus (tingling, neuropathic pain, and significant motor deficits in right-hand flexion and finger movement)
POD 2	Follow-up bronchoscopy: unremarkable trachea, glottis, and bronchial tree
**Ward**	
POD 3	Discharged from ICU
POD 4	Right apical and left basal chest tube and subcutaneous vacuum drain removed
POD 5	Right basal chest tube removed
POD 7	MRI right brachial plexus: mixed pattern of perineural hematoma and edema causing nerve irritation; no structural nerve disruption identified
POD 8	Final chest drain left apical removed (persistent air leak after extensive lung injury)
**Rehabilitation**	
POD 18	Transfer to neurological rehabilitation center
POD 51	Discharge from rehabilitation: marked improvement in shoulder and elbow range of motion; persistent severe distal motor deficit with residual ulnar forearm/hand paresthesia.
3 months	Satisfactory clinical and radiological follow-up; improved arm elevation and hand rotation
ca. 3 years	Electrophysiology: progressive reinnervation of median- and radial-innervated muscles; persistent plegia of ulnar-innervated hand muscles; neuropathic pain controlled with pregabalin

CT, computed tomography; GCS, Glasgow Coma Scale; ICS, intercostal space; ICU, Intensive Care Unit; MRI, magnetic resonance imaging; POD, postoperative day.

## Discussion

The treatment of complex TMI remains challenging – from initial care to definitive surgery. We report a flagpole injury penetrating both lungs and the mediastinum, in close proximity to critical structures such as the aortic arch, trachea, and esophagus. Despite the complexity of the injury, the patient remained conscious and cardiopulmonary stable throughout. This allowed a comprehensive preoperative workup, including CT imaging following careful shortening of the flagpole, esophagoscopy and bronchoscopy, and preparation for potential cardiopulmonary bypass^[^6^]^.

To our knowledge, flagpole-specific bilateral transpulmonary transmediastinal impalement without major sequelae has not been previously described in the indexed literature. Prior reports of impalement-related TMI involving metal bars and pipes describe broadly consistent management principles in hemodynamically stable patients: CT-guided trajectory assessment, endoscopic exclusion of hollow viscus injury, pre-emptive femoral vascular access, and a surgical approach tailored to the object’s trajectory^[^1,6,9,10^]^.

In this case, a FAST scan was omitted, as the patient remained hemodynamically stable for more than 2 hours after the initial trauma, with no clinical signs of cardiac tamponade. CT imaging was performed as the definitive diagnostic modality.

Because of the trajectory between the trachea and the esophagus and uncertain CT findings, we performed an esophagoscopy in the operating room. The sensitivity and specificity of this procedure, when performed by trained endoscopists, exceed 95% for traumatic injuries^[^11^]^. Additionally, fiberoptic bronchoscopy, considered the gold standard for diagnosis, revealed no tracheobronchial injury^[^3^]^.

The patient’s hemodynamic stability permitted a controlled, well-prepared clamshell thoracotomy, an approach routinely performed at our center for lung transplantation. The decision to perform a clamshell thoracotomy rather than a median sternotomy was based on the transmediastinal trajectory of the impaling object and the need for comprehensive exposure of all potentially injured structures. Compared with median sternotomy or bilateral thoracotomy, a clamshell thoracotomy provided superior simultaneous access to both pleural cavities and bilateral pulmonary injuries while maintaining excellent exposure of the mediastinal structures at risk, enabling potentially life-saving maneuvers such as aortic cross-clamping if required^[^6^]^. Thoracoscopic evaluation was not considered appropriate because of the proximity to the aortic arch and bilateral pulmonary involvement, precluding adequate single-lung ventilation.

Throughout the night, trauma management at our institution required substantial resources, including assistance from the fire brigade to shorten the flagpole, and collaboration among several specialized disciplines. We would like to emphasize the importance of carefully stabilizing the flagpole to avoid secondary injuries^[^6^]^. An individual was assigned solely to this task for several hours.

The patient sustained a right-sided inferior brachial plexus injury (C8-T1), most likely caused by the flagpole’s trajectory toward the right superior thoracic aperture, as supported by intraoperative apical parietal pleural contusion and MRI demonstrating perineural edema/hematoma surrounding the right lateral and medial fascicles without nerve discontinuity. The patient had previously undergone microsurgical decompression with right-sided C7/T1 foraminotomy three years prior to the trauma for foraminal stenosis with right C8 radiculopathy – information that was not available at the time of initial assessment – which may have resulted in increased susceptibility to further neural injury. In addition, the patient’s positioning during clamshell thoracotomy may have contributed to or exacerbated the plexus injury alongside the direct traumatic mechanism. Brachial plexopathy is a recognized but uncommon complication in cardiothoracic surgery, most often attributed to excessive chest wall retraction and/or bilateral arm abduction^[^12^]^. Shoulder abduction >90° on arm boards can increase tension on the inferior brachial plexus and represents a potential risk factor, although the patient’s positioning appeared standard (Fig. 2a).

## Conclusion

This case of complex TMI highlights the importance of multidisciplinary trauma management in achieving favorable outcomes and illustrates that stable patients can benefit from comprehensive imaging to guide a safe operative strategy. Continuous stabilization and careful shortening of impaling objects exceeding CT gantry limits are essential to avoid secondary mediastinal injury. Clamshell thoracotomy enabled excellent access to all potentially injured structures.

## Data Availability

Not applicable.

## Additional information

This case was presented as a poster at the Annual Meeting of the Swiss College of Surgeons (Lausanne, Switzerland; June 2024) and at the European Congress of Trauma and Emergency Surgery and World Trauma Congress (Stockholm, Sweden; April 2026).

## Declaration of AI-assisted writing tools

The language refinement of the final manuscript was assisted by the web-based version of DeepL Translator (DeepL SE, Cologne, Germany), an AI-based neural machine translation tool. The web-based DeepL version does not use fixed user-accessible version numbers due to continuous model updates; therefore, no version identifier is reported. The tool was used between summer 2025 and spring 2026. No patient-identifiable data were entered. All outputs were reviewed by J.S., and all authors take full responsibility for the accuracy and integrity of the work.
